# Sex Differences in Cardiovascular Risk Profiles of Patients with Rheumatoid Arthritis: Results from an Italian Multicentre Cohort

**DOI:** 10.3390/jcm13226693

**Published:** 2024-11-07

**Authors:** Fabiola Atzeni, Elena Bartoloni, Fabio Cacciapaglia, Elisa Gremese, Andreina Manfredi, Matteo Piga, Garifallia Sakellariou, Francesca Romana Spinelli, Ombretta Viapiana, Gian Luca Erre

**Affiliations:** 1 Rheumatology Unit, Department of Experimental and Internal Medicine, University of Messina, 98122 Messina, Italy; 2 Rheumatology Unit, Department of Medicine and Surgery, University of Perugia, 06123 Perugia, Italy; elena.bartolonibocci@unipg.it; 3 Rheumatology Unit, Department of Precision and Regenerative Medicine and Ionian Area (DePReMeI), Università degli Studi di Bari, 70124 Bari, Italy; fabio.cacciapaglia79@gmail.com; 4 Department of Medicine and Surgery, “F. De Gennaro” LUM University, Casamassima, 70010 Bari, Italy; 5 Rheumatology and Clinical Immunology, IRCCS Humanitas Research Hospital, 20089 Rozzano, Italy; eli.gremese@gmail.com; 6 Department of Biomedical Sciences, Humanitas University, 20072 Pieve Emanuele, Italy; 7 Rheumatology Unit, Azienda Ospedaliera Universitaria Policlinico of Modena, 41125 Modena, Italy; andreina.manfredi@gmail.com; 8 Rheumatology Unit, AOU Cagliari, Department of Medical Sciences and Public Health, University of Cagliari, 09124 Cagliari, Italy; matteopiga@unica.it; 9 Department of Internal Medicine and Therapeutics, University of Pavia, 27100 Pavia, Italy; filiciasak@gmail.com; 10 Istituti Clinici Scientifici Maugeri IRCCS Pavia, 27100 Pavia, Italy; 11 Rheumatology Unit, Department of Clinical, Internal, Anesthesiological and Cardiovascular Sciences, Sapienza University of Rome, 00185 Rome, Italy; francescaromana.spinelli@uniroma1.it; 12 Rheumatology Unit, Department of Medicine, University and Azienda Ospedaliera Universitaria Integrata of Verona, 37126 Verona, Italy; ombretta.viapiana@univr.it; 13 Rheumatoloy Unit, Department of Medicine, Surgery and Pharmacy, Azienda Ospedaliero-Universitaria of Sassari Italy, 07100 Sassari, Italy; glerre@uniss.it

**Keywords:** rheumatoid arthritis, traditional risk factors, sex, gender, cardiovascular risk scores

## Abstract

**Objective:** The effect of sex and gender-related variables on the evaluation of cardiovascular (CV) risk in rheumatoid arthritis patients has been poorly explored. We investigated the differences in CV risk features and scores according to sex in a wide rheumatoid arthritis (RA) cohort. **Methods:** This is a cross-sectional analysis of a consecutive RA cohort. Disease-specific clinical and serologic variables, traditional CV risk factors and the 10-year CV risk calculated by the SCORE-2, Progetto CUORE and Expanded Risk Score-RA algorithms were compared in males and females. **Results:** A total of 820 patients (193 men, 627 women) were included. Disease activity was similar between the two sexes. A significantly higher prevalence of traditional CV risk factors and higher mean CV risk scores were detected in male compared to female patients. In the multiple linear regression analysis, a higher HAQ, csDMARD use and ACPA positivity were significantly associated with an increased CV risk in females, while b/tsDMARDs was associated with a lower CV risk in males according to different algorithms. **Conclusions:** The distribution of traditional CV risk factors and the 10-year risk of CV disease significantly differed in female and male patients despite similar disease activity. Disease-specific variables may contribute differently to CV risk according to sex. The CV screening in RA should also take into account the different distribution of CV risk factors between sexes.

## 1. Introduction

Rheumatoid arthritis (RA) is an inflammatory disorder that significantly affects patients’ quality of life due to its chronic course and associated multiple comorbidities. In fact, despite the recent introduction of advanced therapies, RA patients still experience a worse functional status and higher mortality risk [1]. It is clear that cardiovascular (CV) comorbidities currently represent one of the most important factors contributing to reduced life expectancy in these patients, partly because the pathogenic mechanisms associated with atherosclerosis acceleration and the effect of CV-preventive therapeutic measures have not been completely ascertained [2]. Nevertheless, growing evidence suggests that the association between RA and CV disease is multifactorial, with various interacting mechanisms linking the two conditions [3]. Among these, traditional CV risk factors, including hypertension, diabetes mellitus and dyslipidemia, play a significant role in accelerating atherosclerosis and CV disease in these patients [4,5]. Moreover, disease-related autoimmune features, chronic inflammation, socioeconomic, ethnic and demographic factors, such as age and sex, may also affect CV morbidity in RA [1]. In fact, RA is about 2–3 times more frequent in women, with data suggesting a progressive increase in disease incidence in females in recent decades [6]. Sex- and gender-related differences in immune responses, as well as hormonal and genetic and environmental factors, surely contribute to different disease expressions, comorbidities, treatment responses, extra-articular manifestations and disease courses in these patients [7]. Similarly, CV comorbidities behave differently in male and female RA patients, although data are highly conflicting due to disparities in the inclusion criteria, environmental exposures and geographical background between studies [8]. Globally, men with RA seem to be characterized by a higher prevalence of traditional CV risk factors and ischemic heart disease, while an increased prevalence of cerebrovascular disease, heart failure with preserved ejection fraction and mortality in CV disease has been reported more often in females [2,8,9]. However, the different effect of sex on the risk of atherosclerosis and overt CV disease in RA patients is difficult to quantify, as several sex- and gender-related traditional and disease-related variables may interfere with a reliable CV risk estimation. Interestingly, in a recent prospective study, the underestimation of CV risk assessed by the EULAR-modified “Systematic COronary Risk Evaluation” (SCORE) algorithm was higher in female in comparison to male RA patients, suggesting that modifying the weight of current algorithms according to sex may improve the estimation of CV risk in RA [10]. However, to date, no study has compared the CV risk using different algorithms in male and female RA patients. Therefore, in the present study, we aimed to compare the features of CV risk factors in a cohort of RA patients according to sex and to compare the CV risk in both groups using different algorithms.

## 2. Methods

This study is a cross-sectional analysis of a cohort of consecutive RA patients fulfilling the 2010 American College of Rheumatology (ACR)/EULAR classification criteria [11] and recruited as of 2019 in 10 Italian Rheumatologic Centers of the “Cardiovascular Obesity and Rheumatic DISease (CORDIS)” Study Group [12]. The following demographic and clinical data were collected: age, sex, smoking status, body mass index (BMI), systolic and diastolic blood pressure values, history of hypertension, use of hypertensive drugs and history of diabetes mellitus (defined based on previous medical history and/or the use of oral or parenteral hypoglycaemic medications or insulin). Ongoing anti-rheumatic drugs, including conventional synthetic (cs) disease-modifying anti-rheumatic drugs (csDMARDs), biologic (b) and targeted synthetic (ts) DMARDs (b/tsDMARDs), and glucocorticoids were recorded. Serologic variables included fasting glucose, total cholesterol (TC), high-density lipoprotein cholesterol (HDL-c), low-density lipoprotein cholesterol (LDL-c) and triglycerides (TG), C reactive protein (CRP), rheumatoid factor (RF) and anti-citrullinated peptide antibodies (ACPA). At baseline, disease activity was evaluated by using the Clinical Disease Activity Index (CDAI) and disability by the Italian version of the Health Assessment Questionnaire (HAQ). The 10-year CV risk was calculated using the SCORE-2, the Progetto CUORE and the Expanded Risk Score in RA (ERS-RA) algorithms. Risk assessment tools that incorporate traditional risk factors (age, sex, smoking, systolic blood pressure, TC and [HDL-c/LDL-c], diabetes mellitus) were used; in addition, as ERS-RA, RA-specific variables were also included [13,14,15]. A multiplication factor of 1.5 was utilized for the SCORE-2 and Progetto CUORE scores in accordance with the EULAR recommendations, given that RA is not included in these algorithms as a separate item [16].

The present study was conducted according to the ethical guidelines of the Declaration of Helsinki and was approved by the local Ethical Committee as part of the GISEA Registry protocol (approval number DG-624/2012–15 March 2012). At the start of the observational period, written informed consent was obtained from all patients.

## 3. Statistical Analysis

Continuous and dichotomous variables were analyzed using the *t*-test for independent samples and the chi-square test, respectively. Appropriately, data are reported as mean ± standard deviation and a number with respective percentages. Multiple linear regression analysis was performed to analyze the linear correlation between RA-specific variables and the CV risk scores for each sex, and the results are expressed as a coefficient and 95% confidence interval (95% CI). Given the relatively high number of missing (missing completely at random, MCAR) data (CRP = 385; RF = 40; ACPA = 46; b/tsDMARD = 1), regression analysis was also performed using multiple imputation (10 sets). Regression models were not adjusted for the independent variables already included in the CV risk score algorithms. The analyses were performed using Stata 18 software (StataCorp, College Station, TX, USA). A *p*-value < 0.05 was considered significant.

## 4. Results

A total of 820 RA patients (193 men and 627 women) with a mean age of 57.6 ± 7.2 years and a median disease duration of 143.8 ± 105.4 months were included. Overall, 65.1% and 67.5% of patients were ACPA and RF-positive, respectively. The complete demographic patient characteristics are shown in Table 1.

Despite similar disease activity, RA male patients had a shorter disease duration (122.6 ± 92.5 vs. 150.3 ± 108.2 months, *p* = 0.001), received csDMARDs more frequently (77.2% vs. 67.9%, *p* = 0.014) and were less frequently ACPA-positive (56% vs. 68%, *p* = 0.003) than their female counterparts. Moreover, female patients had higher disability levels, measured through the HAQ. Treatment with b/tsDMARDs and glucocorticoids was similar between groups.

The distribution of traditional CV risk factors according to sex is illustrated in Table 2. Interestingly, male patients were significantly more frequently active smokers, were affected by diabetes mellitus, had a higher BMI and had higher mean systolic and diastolic blood pressure values at inclusion in comparison to females. However, the prevalence of hypertension (54.9% versus 47.2%, *p* = 0.061) and anti-hypertensive drug use (39.8% versus 34.7%, *p* = 0.243) was similar between the two groups. It was notable that, despite hypertension being detected in 49% of patients in the whole cohort, only one third was on anti-hypertensive treatment.

The analysis of the lipid concentrations profile revealed that female patients had a significantly higher TC (207.9 ± 35.7 mg/dL vs. 199.8 ± 34.6 mg/dL, *p* = 0.005) and HDL-c concentration (59.6 ± 11.4 mg/dL vs. 51.9 ± 11.6 mg/dL, *p* < 0.0001) compared to males.

Finally, the 10-year risk of CV disease was calculated by the SCORE-2, Progetto CUORE and ERS-RA algorithms. As expected, considering the significant differences in the distribution of conventional CV risk factors, the mean CV risk scores were significantly higher in males compared to female patients, as reported in Table 2.

We then performed multiple linear regression analysis in female and male patients, separately. In female RA patients, higher HAQ levels and the use of csDMARDs were significantly associated with an increased CV risk score, calculated by using the Progetto Cuore [coefficient 0.008 (95% CI 0.001 to 0.015); *p* = 0.011], while ACPA positivity was associated with a higher CV risk score, estimated with ERS-RA [coefficient 0.018 (95% CI 0.001 to 0.036); *p* = 0.033] (Table 3).

In contrast, in the regression analysis of male RA patients, b/tsDMARDs use was significantly associated with lower CV risk scores in Progetto Cuore [coefficient −0.014 (95% CI −0.02 to −0.003) *p* = 0.008]. The use of b/tsDMARDs was significant in the SCORE2 algorithm in the listwise analysis [coefficient −0.006 (95% CI −0.011 to −0.0016) *p* = 0.010] and showed a trend towards significance in the computation analysis [coefficient −0.003 (95% CI −0.008 to 0.0002) *p* = 0.06] (Table 4).

## 5. Discussion

In our large cohort of patients with long-standing and moderately active RA, the distribution of traditional CV risk factors differs between males and females, despite the presence of similar disease activity indices. In particular, male patients more frequently show smoking habits and diabetes mellitus and higher BMI systolic and diastolic blood pressure values compared to females at inclusion. Among the serological variables, female RA patients had significantly higher TC and HDL-c concentrations compared to males at inclusion.

These results are in line with findings from different studies showing that male sex is a risk factor for CV disease in RA, likely due to the protective effects of female hormones on endothelial function and atherosclerotic coronary disease in the pre-menopausal phase or the higher prevalence of traditional CV risk factors or atherosclerosis in males [2,17,18,19,20].

The reliable prediction of CV risk in RA patients is still poorly achievable, as traditional algorithms perform lower in these patients in comparison with the general population, even when inflammatory parameters or disease activity are included [21,22]. Indeed, the inflammatory status, beyond conventional and RA-specific risk factors, represents an independent factor that strongly contributes to enhancing the 10-year risk of CV events in these patients [23]. Moreover, the variables included in CV risk algorithms, such as age and sex, have been shown to exert a different impact on estimating the CV disease risk in RA patients compared to the general population. A Dutch study showed that the incidence of CV disease in female RA patients initially classified as low risk was higher than that predicted by using the SCORE algorithm [10]. In fact, sex may influence the clinical expression of CV disease and atherosclerosis in patients with inflammatory joint diseases. However, the varying prevalence of these diseases between sexes, along with a variable severity, makes it difficult to draw definitive conclusions [8]. In our cohort of RA patients, male patients were characterized by a significantly higher risk of future CV events according to three different algorithms in comparison to their female counterparts, in line with the significantly higher prevalence of some traditional CV risk factors, such as smoking, diabetes mellitus, and higher blood pressure values, at inclusion. However, in the study by Crowson et al., despite differences in the prevalence of many CV risk factors between sexes, the relative risk of CV disease did not differ between men and women [9]. Furthermore, the difference in CV event rates between sexes was independent of traditional CV risk factors and markers of disease activity [9]. This suggests that other, not easily measurable, sex-related mechanisms may contribute to the differing CV disease risk in men and women with RA.

In a cross-sectional study, HDL-c subfractions, such as the atheroprotective HDL3- and HDL2-c, were reduced in female and not in male RA patients; in addition, early menopause has been suggested to exert an adverse effect related to sex hormones on the CV risk in female RA patients [24,25]. Moreover, the concomitant use of methotrexate has been associated with a lower risk of major CV events in female patients but not in males [26]. These putative factors may explain, at least in part, why the CV disease risk is underestimated, especially in female RA patients [10]. In this context, the evidence from our cohort showing that CV risk scores, assessed using three different algorithms, performed differently in female and male patients further highlights the varying impact of sex on CV risk estimation in RA patients. In particular, when considering individual algorithms, the 10-year risk of CV disease was higher in women when using the ERS-RA score, while in men, it was higher when using SCORE-2, an algorithm that incorporates sex-specific risk prediction models. This suggests that the disease-specific variables included in ERS-RA may influence CV risk differently in male and female patients. Accordingly, we identified the specific factors significantly associated with the CV risk score that were different in female RA patients compared to their male counterparts. In females, higher disability, ACPA positivity, and the use of csDMARDs were associated with an increased risk of future CV disease, while the use of b/tsDMARDs significantly lowered the CV risk in males. Differences between men and women in disease activity and other RA-specific measures may reflect sex and gender differences in specific parameters, rather than the disease activity itself. Indeed, compared to males, females tend to have higher erythrocyte sedimentation rates and poorer scores on patient-reported outcomes [27]. Similar to other literature data, in our cohort, female patients were characterized by a higher disability index in comparison to males. A lower grip force, higher disease activity, higher prevalence of widespread pain and poorer scores on questionnaires for self-reported health-related items may be associated with a poorer functional status in women [7,28,29,30]. Moreover, we confirmed ACPA positivity as a significant risk factor for the induction and acceleration of atherosclerosis in RA patients [31,32]. Interestingly, this is particularly evident in female patients, suggesting that sex-specific factors may contribute to the pathogenic role of ACPA in the induction of CV disease risk in RA and, in consequence, a more careful CV screening particularly in ACPA-positive females with RA.

Finally, empirical evidence pointed out that multifactorial sex and gender-related variables, as well as differences in drug pharmacokinetics and pharmacodynamics, may influence therapy efficacy in male and female patients [7]. Interestingly, in our cohort, the favorable effect of b/tsDMARD therapy on CV risk was more evident in males, which may reflect their better response to biologic therapy and the higher rate of remission demonstrated in male patients in comparison to their female counterparts [33].

This study has some limitations. We acknowledge that the cross-sectional design of this study may represent a limit of the analysis and that longitudinal observations with long-term outcomes, including mortality, might provide a more accurate picture of the impact of sex on the CV risk in the RA population. Moreover, RA features, such as disease activity, were measured at a single time point, potentially underestimating the impact of RA on CV disease risk. It is also conceivable that other factors not included in the analysis (e.g., dysmetabolic syndrome, insulin resistance, physical activity, diet, antipsychotic drugs) may have a differential impact on cardiovascular risk according to the sex of RA patients.

However, we included a large sample size of consecutive patients, representative of different geographical settings and with homogenous and pre-specified variables, thus reducing selection bias.

To our knowledge, this is the first study to explore three different CV risk algorithms in a wide RA cohort grouped according to sex and the specific factors associated with CV risk in each group. In this setting, it is conceivable that each algorithm may have a different performance if applied to male or female RA patients, and further studies are needed to validate the most reliable algorithm to estimate the CV disease risk in the two sexes and, consequently, tailor the management of the disease according to available recommendations [34].

## 6. Conclusions

In conclusion, sex contributes to generating important epidemiologic, pathogenic and clinical differences in the disease between male and female RA patients. Similarly, it may exert different weights in the assessment of CV disease risk according to international algorithms. According to our findings, the CV screening strategy in RA should also take into account the different distribution of CV risk factors between sexes.

## Figures and Tables

**Table 1:** Clinical features of RA patients according to sex.

	Overall Population n = 820	Females n = 627	Males n = 193	*p* Value
Disease duration, months	143.8 ± 105.4	150.3 ± 108.2	122.6 ± 92.5	**0.001**
Disease duration > 10 yrs, n (%)	447 (54.1)	356 (56.7)	91 (47.1)	**0.019**
CRP, mg/L	6.3 ± 9.3	6.3 ± 9.7	6.0 ± 8.1	0.73
RF positivity, n (%) °	536 (67.5)	416 (68.5)	120 (64.1)	0.26
ACPA positivity, n (%) *	514 (65.1)	408 (68.0)	106 (56.0)	**0.003**
CDAI	9.2 ± 9.5	9.2 ± 9.2	9.0 ± 10.2	0.71
HAQ	0.72 ± 0.75	0.80 ± 0.77	0.47 ± 0.61	**<0.0001**
Glucocorticoid use, n (%)	352 (42.9)	272 (43.3)	80 (41.4)	0.63
csDMARDs use, n (%)	575 (70.1)	426 (67.9)	149 (77.2)	**0.014**
b/tsDMARDs use, n (%)	459 (55.9)	356 (56.7)	103 (53.3)	0.40

Values are mean ± SD, or n (%); CRP, C-reactive protein; RF, rheumatoid factor; ACPA, anti-citrullinated peptide antibodies; CDAI, clinical disease activity index; HAQ, Health Assessment Questionnaire; csDMARDs, conventional synthetic disease-modifying anti-rheumatic drugs; b/tsDMARDs, biologic or targeted disease-modifying anti-rheumatic drugs. * available on 789 RA patients; ° available on 734 RA patients. Statistically significant differences are in **bold**.

**Table 2:** Cardiovascular risk factor distribution in RA patients according to sex.

	Overall RA Population n = 820	Females n = 627	Males n = 193	*p* Value
Age, years	57.6 ± 7.2	57.5 ± 7.3	58.0 ± 7.0	0.46
BMI, kg/m^2^	25.9 ± 4.6	25.7 ± 4.8	26.6 ± 3.8	**0.015**
Total cholesterol, mg/dL	206.0 ± 35.6	207.9 ± 35.7	199.8 ± 34.6	**0.005**
HDL cholesterol, mg/dL	57.8 ± 11.9	59.6 ± 11.4	51.9 ± 11.6	**<0.0001**
Triglycerides, mg/dL	108.4 ± 51.9	106.6 ± 51.9	114.2 ± 51.5	0.07
Hyperlipidemia, n (%)	453 (55.2)	364 (58.0)	89 (46.1)	**0.004**
Diastolic blood pressure, mmHg	76.9 ± 9.7	76.2 ± 9.9	79.0 ± 8.8	**<0.001**
Systolic blood pressure, mmHg	126.4 ± 16.0	125.5 ± 16.4	129.5 ± 14.4	**0.002**
Hypertension, n (%)	402 (49.0)	296 (47.2)	106 (54.9)	0.061
Use of antihypertensive drugs, n (%)	294 (35.8)	218 (34.7)	76 (39.8)	0.243
Smoke, n (%)	182 (22.2)	121 (19.3)	61 (31.6)	**<0.001**
Diabetes, n (%)	57 (6.95)	37 (5.9)	20 (10.3)	**0.033**
CUORE *	0.068 ± 0.077	0.045 ± 0.043	0.140 ± 0.112	**<0.001**
SCORE-2 *	0.075 ± 0.047	0.064 ± 0.038	0.110 ± 0.053	**<0.001**
ERS-RA score	0.105 ± 0.084	0.089 ± 0.066	0.155 ± 0.112	**<0.001**

Values are median ± 1SD. * The score has been adapted by a multiplication factor of 1.5 according to EULAR recommendations. Statistically significant differences are in **bold**.

**Table 3:** RA-related factors influencing CV risk scores in female RA patients.

	Listwise Analysis (n = 417)				Imputation Analysis (n = 627)				
	Coeff.	95% CI		*p*-Value		Coeff.	95% CI		*p*-Value
**CUORE**					**CUORE**		n = 627		
Disease duration > 10 yrs	−0.0018	−0.0106	0.0070	0.686	Disease duration > 10 yrs	0.0013	−0.0057	0.0083	0.716
CDAI	−0.0004	−0.0010	0.0000	0.079	CDAI	−0.0002	−0.0007	0.0001	0.189
HAQ	0.0086	0.0019	0.0152	**0.011**	HAQ	0.0087	0.0033	0.0140	**0.001**
Steroid	−0.0002	−0.0091	0.0086	0.961	Steroid	−0.0051	−0.0124	0.0020	0.162
csDMARDs	0.0035	−0.0002	0.0073	0.064	csDMARDs	0.0044	0.0013	0.0075	**0.005**
btsDMARD	−0.0011	−0.0035	0.0012	0.340	btsDMARD	0.0001	−0.0016	0.0018	0.14
CRP	0.0000	−0.0004	0.0004	0.961	CRP	0.0001	−0.0003	0.0005	0.617
ACPA	0.0046	−0.0065	0.0159	0.414	ACPA	0.0041	−0.0049	0.0132	0.374
RF	0.0063	−0.0050	0.0177	0.272	RF	0.0062	−0.0029	0.0153	0.182
**SCORE2**					**SCORE2**		n = 627		
Disease duration > 10 yrs	−0.0044	−0.0121	0.0032	0.259	Disease duration > 10 yrs	−0.0019	−0.0082	0.0043	0.535
CDAI	−0.0003	−0.0007	0.0001	0.168	CDAI	−0.0000	−0.00047	0.0003	0.684
HAQ	0.0055	−0.0002	0.0113	0.059	HAQ	0.0050	0.0002	0.0097	0.037
Steroid	0.0017	−0.0060	0.0094	0.666	Steroid	−0.0029	−0.0094	0.0034	0.368
csDMARDs	0.0032	−0.0000	0.0065	0.057	csDMARDs	0.0037	0.0009	0.0064	**0.008**
btsDMARD	−0.0020	−0.0040	0.0000	0.057	btsDMARD	−0.0005	−0.0020	0.0010	0.520
CRP	7.17 × 10^−7^	−0.0003	0.0003	0.997	CRP	0.0000	−0.0002	0.0004	0.681
ACPA	0.0053	−0.0044	0.0151	0.286	ACPA	0.0042	−0.0039	0.0124	0.305
RF	0.0041	−0.0057	0.0141	0.407	RF	0.0049	−0.0031	0.0131	0.231
**ERSRA**					**ERSRA**		n = 627		
csDMARDs	0.0041	−0.0017	0.0100	0.169	csDMARDs	0.0045	−0.0001	0.0092	0.056
btsDMARD	−0.0033	−0.0069	0.0003	0.073	btsDMARD	−0.0023	−0.0049	0.0002	0.072
CRP	0.0003	−0.0003	0.0009	0.368	CRP	0.0004	−0.0002	0.0010	0.215
ACPA	0.0188	0.0015	0.0361	**0.033**	ACPA	0.0143	0.0004	0.0282	**0.043**
RF	0.0028	−0.0148	0.0204	0.756	RF	0.0071	−0.0066	0.0209	0.307

CDAI, clinical disease activity index; HAQ, Health Assessment Questionnaire; csDMARDs, conventional synthetic disease-modifying anti-rheumatic drugs; btsDMARDs, biologic or targeted disease-modifying anti-rheumatic drugs; CRP, C-reactive protein; ACPA, anti-citrullinated peptide antibodies; RF, rheumatoid factor. Statistically significant differences are in **bold**.

**Table 4:** RA-related factors influencing CV risk scores in male RA patients.

	Listwise Analysis (n = 143)				Imputation Analysis (n = 193)				
	Coefficient	95% CI		*p*-Value		Coefficient	95% CI		*p*-Value
**CUORE**					**CUORE**				
Disease duration > 10 yrs	0.0337	−0.0050	0.0725	0.088	Disease duration > 10 yrs	0.0152	−0.0186	0.0490	0.376
CDAI	−0.0002	−0.0023	0.0018	0.781	CDAI	−0.0000	−0.0019	0.0017	0.950
HAQ	0.0186	−0.0186	0.0559	0.324	HAQ	0.0044	−0.0275	0.0364	0.782
Steroid	−0.0233	−0.0661	0.0194	0.282	Steroid	−0.0048	−0.0408	0.0311	0.791
csDMARDs	0.0037	−0.0134	0.0209	0.669	csDMARDs	0.0012	−0.0134	0.0159	0.864
btsDMARD	−0.0147	−0.0255	−0.0039	**0.008**	btsDMARD	−0.0097	−0.0184	−0.0009	**0.030**
CRP	0.0002	−0.0020	0.0026	0.805	CRP	0.0004	−0.0018	0.0027	0.713
ACPA	0.0242	−0.0233	0.0718	0.315	ACPA	0.0128	−0.0288	0.0545	0.544
RF	0.0099	−0.0386	0.0585	0.687	RF	0.0106	−0.0322	0.0536	0.624
**SCORE2**					**SCORE2**				
Disease duration > 10 yrs	0.0078	−0.0100	0.0256	0.388	Disease duration > 10 yrs	−0.0018	−0.0179	0.0142	0.823
CDAI	−0.0003	−0.0012	0.0006	0.497	CDAI	−0.0002	−0.0010	0.0006	0.630
HAQ	0.0116	−0.0054	0.0288	0.180	HAQ	0.0063	−0.0088	0.0215	0.410
Steroid	−0.0170	−0.0367	0.0026	0.089	Steroid	−0.0100	−0.0272	0.0070	0.247
csDMARDs	0.0005	−0.0073	0.0084	0.888	csDMARDs	−0.0002	−0.0071	0.0067	0.954
btsDMARD	−0.0065	−0.0115	−0.0016	**0.010**	btsDMARD	−0.0039	−0.0080	0.0002	0.064
CRP	0.0007	−0.0003	0.0018	0.203	CRP	0.0006	−0.0004	0.0018	0.217
ACPA	0.0021	−0.0198	0.0240	0.848	ACPA	−0.0036	−0.0234	0.0161	0.717
RF	0.0062	−0.0161	0.0285	0.583	RF	0.0085	−0.0119	0.0290	0.413
**ERSRA**					**ERSRA**				
csDMARDs	−0.0028	−0.0187	0.0131	0.728	csDMARDs	−0.0005	−0.0150	0.0138	0.938
btsDMARD	−0.0067	−0.0163	0.0029	0.172	btsDMARD	−0.0038	−0.0122	0.0044	0.361
CRP	0.0021	−0.0000	0.0043	0.052	CRP	0.0019	−0.0002	0.0040	0.085
ACPA	0.0177	−0.0268	0.0624	0.432	ACPA	0.0055	−0.0355	0.0467	0.789
RF	0.0196	−0.0258	0.0651	0.394	RF	0.0175	−0.0245	0.0596	0.412

CDAI, clinical disease activity index; HAQ, Health Assessment Questionnaire; csDMARDs, conventional synthetic disease-modifying anti-rheumatic drugs; btsDMARDs, biologic or targeted disease-modifying anti-rheumatic drugs; CRP, C-reactive protein; ACPA, anti-citrullinated peptide antibodies; RF, rheumatoid factor. Statistically significant differences are in **bold**.

## Data Availability

The data supporting the results of this study are available from the corresponding Author upon reasonable request.
